# *RET* rearrangement-positive pancreatic cancer has remarkable response to pralsetinib: a case report

**DOI:** 10.3389/fonc.2023.1078076

**Published:** 2023-04-17

**Authors:** Tongyi Zhang, Hongwei Wang, Zhiwei Cai, Siqi Zhang, Chongyi Jiang

**Affiliations:** ^1^ Department of General Surgery, Huadong Hospital, Fudan University, Shanghai, China; ^2^ Department of Hepato-Biliary-Pancreatic Surgery, Huadong Hospital, Fudan University, Shanghai, China; ^3^ The Medical Department, 3D Medicines Inc., Shanghai, China

**Keywords:** pancreatic cancer, RET fusion, pralsetinib, target therapy, RET inhibitors

## Abstract

Patients with metastatic pancreatic cancer have limited treatment options and a dismal prognosis. While *RET* fusion is rare (0.6%) in pancreatic cancer, the efficacy of RET-targeted treatment in patients with *TRIM33-RET* fusion has not been previously reported. Herein, we presented a case of a 68-year-old man with pancreatic cancer harboring *TRIM33-RET* fusion who responded remarkably to pralsetinib despite being intolerant to chemotherapy. To our knowledge, this is the first report on the clinical value of a single *TRIM33-RET* fusion in pancreatic cancer, which may benefit from the targeted therapy.

## 1 Introduction

Pancreatic cancer has a high degree of malignancy and rapid progression. According to the latest statistics from the China National Cancer Center, the annual incidence of pancreatic cancer in China is approximately 4.29/100,000, a considerable increase from 15 years ago (1). For all stages combined, the 5-year survival rate was 5%–10% (2, 3). Chemotherapy remains the primary treatment for pancreatic cancer. However, the progression-free survival (PFS) for first-line chemotherapy in advanced pancreatic cancer patients is typically approximately 3–6 months (4). The advancement of targeted therapy has increased the number of potential benefits. Olaparib has been approved by the Food and Drug Administration (FDA) as a first-line maintenance treatment for metastatic pancreatic cancer patients with germline *BRCA1/2* mutations based on the improvement in progression-free survival demonstrated in a randomized phase III POLO trial (5). Larotrectinib and entrectinib have been approved as agnostic treatments for solid malignancies with *NTRK* fusion (6, 7). Patients harboring the *NRG1* gene fusion are sensitive to Zenocutuzumab (8). The activity of adagrasib and sotorasib in *KRAS G12C* pancreatic cancer also provides new hope for *KRAS*-mutant pancreatic cancer patients (9, 10). However, these drug-targeted genetic mutations only account for a low percentage of pancreatic cancer cases. Therefore, it is essential to search for precision therapies based on genetic alterations for pancreatic cancer patients.

The proto-oncogene *RET* encodes a membrane receptor tyrosine kinase involved in many cellular processes, including the development of the central nervous system, peripheral nervous system, and kidney (11, 12). *RET* fusions are activated in a ligand-independent manner, promoting cancer cell proliferation and survival (13). As a result, RET fusion proteins have become an attractive target for precision medicine. RET inhibitors, such as selpercatinib and pralsetinib, have demonstrated efficacy in patients with *RET* fusion-positive tumors. The incidence of *RET* fusion in pancreatic cancer is 0.6% (14).

In this case, a *TRIM33-RET* fusion was detected through next-generation sequencing (NGS) in a patient with pancreatic ductal adenocarcinoma (PDAC) who responded well to pralsetinib.

## 2 Case presentation

**Figure 1:**
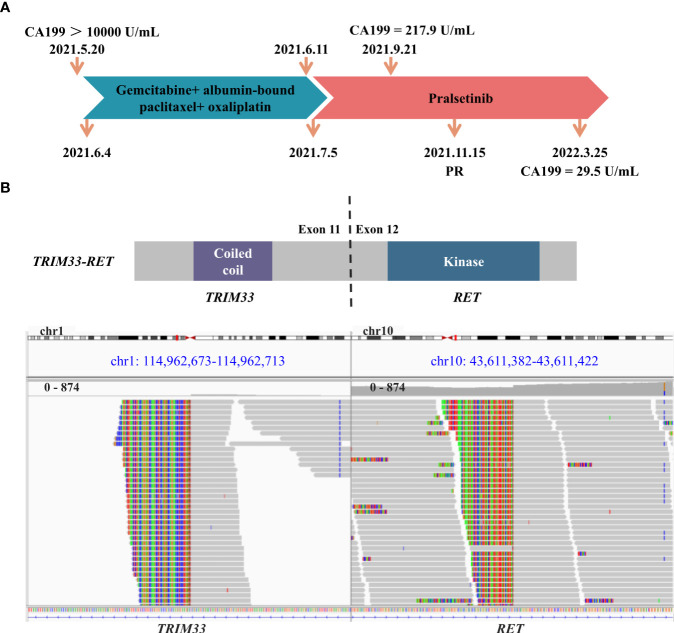
Schematic of treatment history and next-generation sequencing (NGS)‐detected *RET* fusion. **(A)** The timeline of treatment and corresponding CA199 levels. **(B)** The schematic diagram and identification of the *TRIM33-RET* fusion. Sequencing reads of *TRIM33* and *RET* are visualized by the Integrative Genomics Viewer (IGV).

**Figure 2:**
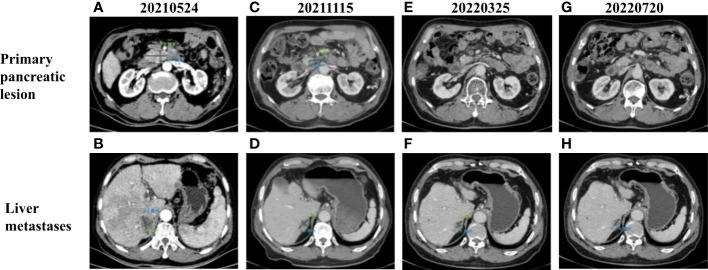
**(A)** CT images of primary pancreatic lesion with a size of 4.1 cm × 2 cm before treatment. **(B)** CT images of liver metastases with a size of 3.8 cm × 2.4 cm before treatment. Contrast-enhanced CT scan images completed in **(C, D)** November 2021, **(E, F)** March 2022, and **(G, H)** July 2022 demonstrating progressive decrease in the size of primary pancreatic and liver metastases.

A 68-year-old man was admitted to our hospital on 20 May 2021 due to persistent upper abdominal pain. The patient had no personal or family history of malignancy, pancreatitis, or liver disease. His serum CA 19-9 level was above 10,000 U/ml (**Figure 1A**), and his carcinoembryonic antigen (CEA) level was 68.7 U/ml. A CT scan revealed a 4.1 × 2.0-cm mass in the pancreatic uncinate process and a 3.8 × 2.4-cm mass in the liver (**Figures 2A, B**). Pathological evaluation of the tissue samples with liver biopsy indicated PDAC. Immunohistochemical (IHC) staining revealed that the cells were positive for CA19-9 and CK7 while negative for p53, CK20, AFP, and c-erbb-2. Ki-67 exhibited a 70% proliferative rate. To explore precision treatment options, a biopsy tissue sample from the patient was sent for NGS analysis using a 733-gene panel. The test was performed by a laboratory (3D Medicine Inc., Shanghai, China) certified by the College of American Pathologists (CAP), Clinical Laboratory Improvement Amendments (CLIA), and China National Accreditation Service for Conformity Assessment (CNAS). The tumor mutational burden (TMB) was 4.47 mutations/Mb, and the microsatellite status was stable. Meanwhile, a somatic *RET* fusion (*TRIM33-RET*) was detected (**Figure 1B**). Moreover, other pathogenic or likely to be pathogenic variations were detected, including somatic *TGFBR1* (p.I66Yfs*9, 23.94%) mutation, amplification of *BCORL1* (copy number = 6), and germline *RAD50* mutation (*RAD50*, p.K722Nfs*6). In addition, the patient was found to have wild-type variants in *HER2*, *BRCA1/2*, and *RAS/RAF*.

Based on the previous clinical research results (15–18), the patient’s financial situation, genetic testing results, and guidelines, first-line chemotherapy (albumin-bound paclitaxel 200 mg/m^2^, oxaliplatin 85 mg/m^2^, and gemcitabine 1.4 g/m^2^) was administered on 4 June 2021. However, the patient rapidly developed significant gastrointestinal toxicity and myelosuppression. Due to intolerance, the patient only had one course of chemotherapy. Since 5 July 2021, the patient received 400 mg of pralsetinib daily. Four months later, the volume of the primary tumor decreased by approximately 39.0% (**Figures 2A, C**), and that of the metastases decreased by approximately 39.5% (**Figures 2B, D**) compared to that before treatment, and a partial response was confirmed. However, due to the adverse effect of anemia in the patient, the treatment dose was reduced accordingly. From 15 November 2021 to the present, the patient has been receiving 200 mg of pralsetinib daily for maintenance therapy. Also, the tumor biomarker cancer antigen CA199 dropped from a very high level (>10,000 U/ml) to 29.5 U/ml (**Figure 1A**). Currently, it was concluded that the patient reached a partial response. The patient’s primary tumor and metastases were still shrinking (**Figures 2E–H**), and the progression-free survival was at least 12 months.

## 3 Discussion

The *TRIM33-RET* fusion protein contains a coiled-coil domain encoded by *TRIM33* exons 1–11 and a complete kinase domain encoded by *RET* exons 12–20, which may result in the activation of the RET tyrosine kinase. *RET* fusion is a rare genomic alteration in the PDAC. *TRIM33-RET* fusion has previously been reported in non-small cell lung cancer and oncocytic intraductal carcinoma of salivary glands (19, 20). We reported on a case of advanced PDAC that responded to pralsetinib as second-line systemic therapy. The PFS has been more than 12 months.

New targeted drugs for *RET* fusion are constantly emerging in succession (21). Recently, selpercatinib and pralsetinib were approved by the FDA for the treatment of lung and thyroid cancers with *RET* gene mutations or fusions (22), (23). Although no targeted drug for *RET* fusion-positive PDAC has been approved, ongoing clinical studies of target drugs for *RET* fusion in more cancer types are underway. The ARROW study is a multi-cohort, open-label, phase 1/2 study designed to investigate pralsetinib for the treatment of *RET*-altered solid tumors, including four patients with pancreatic cancer (24). The results confirmed that response occurred in 57% of 23 evaluable patients (24). The most common grade 3–4 treatment-related adverse events (TRAEs) in the pre-treated population were neutropenia, anemia, and hypertension (24). Notably, *JMJD1C-RET* fusion and *TRIM33-RET* fusion were detected in a pancreatic cancer patient who achieved an ongoing complete response at a treatment duration of 33.1 months (24). The LIBRETTO-001 study, a phase 1/2 study of selpercatinib in participants with advanced solid tumors, *RET* fusion-positive solid tumors, and medullary thyroid cancer, had been reported. Forty-five patients with *RET* fusion had been enrolled, including 12 patients with pancreatic cancer. The overall response rate (ORR) was 43.9% in 41 efficacy-evaluable patients confirmed by an independent review committee (25). Many novel selective RET inhibitors have shown good efficacy and low off-target toxicity in clinical trials (e.g., BLU-667 and LOXO-292), which encourages the development and research of more selective RET inhibitors (10). This is the first case report of a patient with only the *TRIM33-RET* fusion, a single fusion gene, detected who has an ongoing partial response to pralsetinib in PDAC.

## 4 Conclusion

In conclusion, this is the first case report in which a patient with only the *TRIM33-RET* fusion, a single fusion gene, detected in PDAC had a remarkable response to pralsetinib. This suggests the importance of NGS testing for patients with PDAC, especially those intolerant to chemotherapy.

## Data availability statement

The original contributions presented in the study are included in the article/supplementary material. Further inquiries can be directed to the corresponding author.

## Ethics statement

The studies involving human participants were reviewed and approved by The Local Ethics Review Committee. The patients/participants provided their written informed consent to participate in this study. Written informed consent was obtained from the individual(s) for the publication of any potentially identifiable images or data included in this article.

## Author contributions

CJ, TZ, and HW followed up the patient and collected patient data. SZ contributed to the writing of the original draft. ZC contributed to the collection of CT image data. All authors contributed to the article and approved the submitted version.
